# The Emerging Role of HMGB1 in Neuropathic Pain: A Potential Therapeutic Target for Neuroinflammation 

**DOI:** 10.1155/2016/6430423

**Published:** 2016-05-18

**Authors:** Wenbin Wan, Lan Cao, Ramin Khanabdali, Bill Kalionis, Xiantao Tai, Shijin Xia

**Affiliations:** ^1^Department of Neurology, Zhongshan Hospital, Fudan University, Shanghai 200032, China; ^2^State Key Laboratory of Medical Neurobiology, Department of Neurobiology and Institutes of Brain Science, School of Basic Medical Science, Fudan University, Shanghai 200032, China; ^3^Department of Maternal-Fetal Medicine, Pregnancy Research Centre and Department of Obstetrics and Gynaecology, Royal Women's Hospital, University of Melbourne, Parkville, VIC 3052, Australia; ^4^School of Acupuncture, Massage and Rehabilitation, Yunnan University of Traditional Chinese Medicine, Kunming 650500, China; ^5^Shanghai Institute of Geriatrics, Huadong Hospital, Fudan University, Shanghai 200040, China

## Abstract

Neuropathic pain (NPP) is intolerable, persistent, and specific type of long-term pain. It is considered to be a direct consequence of pathological changes affecting the somatosensory system and can be debilitating for affected patients. Despite recent progress and growing interest in understanding the pathogenesis of the disease, NPP still presents a major diagnostic and therapeutic challenge. High mobility group box 1 (HMGB1) mediates inflammatory and immune reactions in nervous system and emerging evidence reveals that HMGB1 plays an essential role in neuroinflammation through receptors such as Toll-like receptors (TLR), receptor for advanced glycation end products (RAGE), C-X-X motif chemokines receptor 4 (CXCR4), and N-methyl-D-aspartate (NMDA) receptor. In this review, we present evidence from studies that address the role of HMGB1 in NPP. First, we review studies aimed at determining the role of HMGB1 in NPP and discuss the possible mechanisms underlying HMGB1-mediated NPP progression where receptors for HMGB1 are involved. Then we review studies that address HMGB1 as a potential therapeutic target for NPP.

## 1. Introduction

Pain is a type of physical experience defined as an unpleasant sensory and mental problem resulting from actual or potential tissue damage, or something that makes people uncomfortable (International Association for the Study of Pain) [1]. Thus, pain is a multidimensional and subjective experience. Peripheral tissue injury or inflammation can cause reversible adaptive changes in the sensory nervous system, such as hyperalgesia, that provide a protective role against further nociceptive stimuli, leading to the promotion of wound healing and the subsidence of inflammation [2], while neuropathic pain (NPP) is intolerable, persistent, and specific type of long-term pain. NPP is considered to be a direct consequence of pathological changes affecting the somatosensory system and can be debilitating in affected patients [3, 4]. NPP is relatively common, occurring in about 8% of the population, and can impact on the patient's health and many aspects of their quality of life [5, 6]. In the USA, pain severity in patients suffering from NPP significantly correlated with NPP-related issues such as health care resource utilization, personal productivity, and costs [7].

NPP is not a disease, but a syndrome manifested by common and less common signs and symptoms [8]. NPP may originate from the central nervous system (CNS) or periphery and is characterized by both spontaneous and provoked pain, as well as by paresthesia, dysesthesia, and deficits in normal sensation reflecting nerve damage [9]. An innocuous irritation can lead to pain, but it has been determined that the duration and the extent of the response to stimuli can magnify the pain, indicating that the threshold of NPP falls dramatically with pain progression [10]. In general, NPP has a peripheral origin, arising as a consequence of peripheral nerve injury or as a consequence of a metabolic disease such as diabetes [11]. However, NPP can also be caused by infectious diseases such as postherpetic neuralgia and can be manifested in disorders of various etiologies such as spinal cord injury, local poststroke ischemia, cancer, and complex regional pain syndrome [1, 4, 12, 13] (Table 1).

Accumulating evidence indicates that NPP is not merely a concept, or a single disorder, or neural tissue damage caused by a simple nociception. Indeed NPP is considered to be an evolving collection of established clinical and experimental conditions that share the prolongation of manifested pain symptoms or pain-related behaviors [24]. Abnormalities in neural activity predictably play essential roles in the spontaneous pain typical of NPP states [24]. Although the pathogenesis of NPP has been intensively studied in recent decades, the underlying mechanisms remain to be clarified and currently there are no effective therapies for NPP. Thus, identification of the novel molecular machinery involved in NPP may lead to the development of promising analgesics and is therefore eagerly anticipated.

Recently, high mobility group protein box 1 (HMGB1) was identified as an important inflammatory mediator in various diseases caused by an abnormally enhanced inflammatory immune response [25]. Furthermore, mounting evidence supports the hypothesis that HMGB1 plays an essential role in the pathology of NPP progression. Here, we discuss the evidence from studies that address the role of HMGB1 in NPP. We firstly review studies concerning the role of HMGB1 in NPP and discuss the possible mechanisms underlying HMGB1-mediated NPP progression. Then, we review the evidence indicating that HMGB1 is a potential therapeutic target for NPP.

## 2. HMGB1

**Figure 1:**
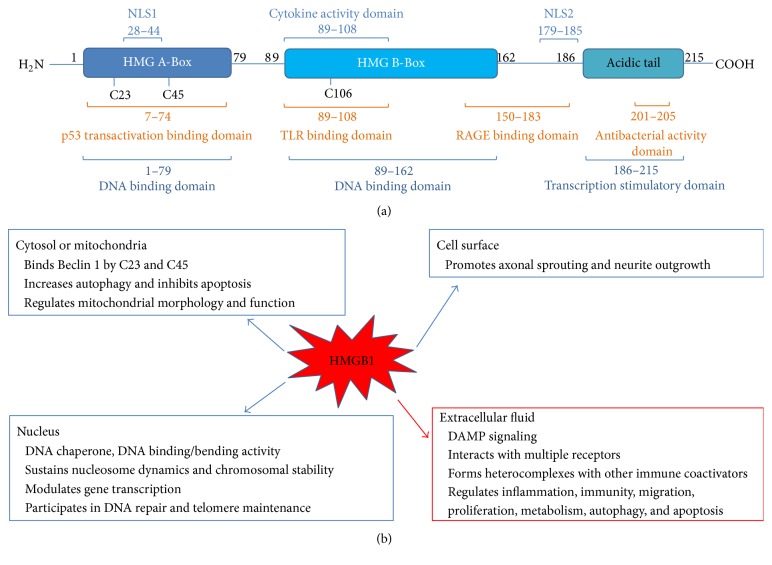
Multiple roles of HMGB-1 in cell response.

In 1973, Goodwin discovered a group of nonhistone nuclear proteins that were characterized by high electrophoretic mobility and called them high mobility group (HMG) proteins. These proteins include three superfamilies designated as HMGB, HMGN, and HMGA [26–28]. HMGB1 (also known as amphoterin) is the most abundant and well-studied HMG protein, which has been highly conserved in evolution and is ubiquitously expressed in most cell types [29–31]. HMGB1 consists of 215 amino acids encoded by a gene on human chromosome 13q12-13. HMGB1 has two positively charged domains (box A and box B) that are involved in binding to the nucleosome structure and govern gene expression via combination with transcription elements (Figure 1(a)).

Investigations revealed that HMGB1 isoforms in different redox states are involved in activating immune cells [32, 33]. These redox states result from posttranslational modifications dependent on three cysteines at positions 23, 45, and 106 (C23, C45, and C106, as shown in Figure 1(a)). HMGB1 interacts with TLR4 via the disulfide bond at C23 and C45 and the free thiol at C106 [33] and in doing so induces cytokine production. In contrast to its inactive form, which contains disulfide bonds at all three sites, HMGB1 does not interact with TLR4 but interacts with CXCL12 to promote chemotaxis [32]. Therefore, the redox modification of HMGB1 is critical in induction of immune responses.

HMGB1 also has a major role as a nonhistone nucleosomal regulatory nuclear protein that is pivotal in DNA rehabilitation and replication [29, 34]. Under physiological conditions, HMGB1 is located in the nucleus where it acts as a DNA chaperone with a role in repair, recombination, regulation of transcription, replication, and genome stability [35]. HMGB1 also has important extracellular functions either through passive release by necrotic cells following cell disruption and nuclear breach or by spontaneous secretion from monocytes and macrophages, where HMGB1 acts as an amplifier of proinflammatory signals originating from innate immune cells [29, 36, 37]. Once located extracellularly, HMGB1 acts as a prototypical damage-associated molecular pattern molecule (DAMP) and promotes inflammasome activation [32]. This particular DAMP cooperates with other factors such as chemokines, growth factors, and cytokines and orchestrates the inflammatory and immune response [38]. Similarly in the immune system, activated immunocytes release HMGB1 into extracellular locations and the details surrounding this process are starting to emerge [39]. However, the mechanism of how other cells, including sensory neurons and spinal glial cells, regulate HMGB1 release in response to different signals remains largely unknown [40] (Figure 1(b)). HMGB1 released by immune cells is highly proinflammatory via cooperation with several definitive receptors that are involved in the inflammatory reaction. These receptors include the receptor for advanced glycation end products (RAGE) [41, 42], Toll-like receptors (TLR-2, TLR-4, and TLR-9) [43–45], integrin [46], SNCA/*α*-Synuclein filaments [47], CD24 [48], and NMDA receptor [49]. Accumulating evidence shows that its role as an “alarmin” is a pivotal biological role for HMGB1 [50]. HMGB1 recruits and stimulates antigen-presenting cells (APC), such as dendritic cells (DCs). Usually, HMGB1 is an alarm signal that denotes endogenous danger and reports cell damage/necrosis as well as the necessity for repair and induction of a “sterile” immune response by boosting both innate and adaptive immune responses [29, 36, 51]. More importantly, HMGB1 in the cytoplasm plays a role as a cytokine when it is secreted by macrophages in case of injury, inflammation, or disease [52].

## 3. HMGB1 in NPP

The first study that revealed the proinflammatory properties of HMGB1 was conducted more than 10 years ago [53], but given the potentially important implications of the study, there are fewer than expected reports on the relationship between NPP and HMGB1. HMGB1 applied to the sciatic nerve in rats via a preimplanted, indwelling perisciatic catheter induced a dose-dependent reduction in mechanical thresholds against von Frey filament stimulation but no apparent thermal hyperalgesia over a period of 24 h after application [53]. Subsequently, a similar result was observed after the application of HMGB1 to the sciatic nerve after blunt dissection to expose the nerve [54]. More importantly, HMGB1 induced pain-like behavior without prior surgery [54]. Subcutaneous injection of HMGB1 to the plantar side of the paw, as well as intra-articular injection of HMGB1 to the ankle joint of mice, drives mechanical hypersensitivity [54].

Recently, HMGB1 was shown to be secreted not only by leukocytes but also by irritated or damaged neurons, which release HMGB1 into the extracellular space [55]. Accumulating evidence indicates that HMGB1 is also involved in the pathology of NPP [2, 27, 40, 42, 56]. In the spinal nerve ligation (SNL) disease model, HMGB1 expression was detected in Schwann cells in the spinal nerve, in the primary afferent neurons, and in satellite glial cells (SGCs) in the dorsal root ganglion (DRG) [57, 58]. HMGB1 is involved in the translocation of a damage signal from the nucleus to the cytoplasm. HMGB1 forms complexes with other factors and enhances the effect of its partner molecules (e.g., IL-1 or LPS), and one unexplored possibility is that HMGB1 directly or indirectly facilitates neuronal activity by also potentiating the actions of RAGE or TLRs [59, 60]. In contrast, an HMGB1 neutralizing antibody inhibits pain onset in NPP models [57, 61]. In a DRG study, treatment with an anti-HMGB1 neutralizing antibody significantly depressed TNF-*α* expression [61]. Furthermore, behavioral tests to investigate the mechanical withdrawal threshold revealed that the administration of anti-HMGB1 neutralizing antibody improved the pain-related behavior [61].

Nuclear HMGB1 immunoreactivity has been detected in various cells such as neurons, satellite cells, Schwann cells, microglia, and astrocytes from naive rodents [57, 62, 63]. Increased levels of both HMGB1 mRNA and protein have been detected in several pain studies, and this has been interpreted as HMGB1 release and involvement in nociception, since these levels are decreased following the administration of an HMGB1 neutralizing antibody [42, 64]. In a study on the involvement of HMGB1 in mechanical allodynia in a model of type 2 diabetes, the development of mechanical allodynia in the rodent was associated with upregulation of HMGB1 protein in the spinal cord [65]. An intrathecal injection HMGB1 neutralizing antibody inhibited mechanical allodynia [65]. The mRNA levels of inflammatory mediators, including interleukin, TNF-*α*, and monocyte chemoattractant proteins, were upregulated together with glial fibrillary acidic protein (GFAP) in the spinal dorsal horn in this model and these effects were attenuated by application of the HMGB1 neutralizing antibody [65]. In another study, intravenous treatment with an anti-HMGB1 monoclonal antibody significantly ameliorated partial sciatic nerve ligation- (PSNL-) induced hind paw tactile hypersensitivity and decreased the injury-induced expression of cFos and iba-1 [63]. These data indicate that the synthesis and secretion of HMGB1 from spinal neurons increase in nerve injury, facilitating the activity of multiple type cells including microglia, astrocytes, and neurons, which in turn results in the symptoms of NPP and suggests that HMGB1 could be a potential therapeutic target for NPP.

## 4. Receptors for HMGB1 in NPP

### 4.1. RAGE

RAGE is a transmembrane cell signaling receptor that belongs to the immunoglobulin superfamily of cell surface multiligand receptors [66, 67]. Mounting evidence reveals that RAGE is a potential contributor to the pathogenesis of many diseases, such as diabetic complications, atherosclerosis, chronic inflammation, cancer, and neurodegeneration [37, 66, 68]. RAGE is widely expressed in human immunocytes, vascular endothelial cells, and neuron and glial cells, but its basal expression is low except in the lungs [37, 69, 70]. RAGE expression leads to the upregulation of proinflammatory factor and cytokines [71]. RAGE was originally identified as a receptor for advanced glycation end products (AGEs). However, RAGE also binds to other structurally multiform ligands, including all-thiol HMGB1, S100 family members, and amyloid-*β* (A*β*), whereby it regulates multiple physiological and pathological processes [66, 67]. By virtue of its multidomain structure and ability to recognize different classes of ligands, RAGE behaves as a pattern recognition receptor (PRR), analogous to innate immune receptors such as TLRs, and plays a role in orchestrating the immune response [72]. In contrast to other PRRs, RAGE not only binds to exogenous ligands but also interacts with endogenous ligands, especially those considered to be DAMPs, such as HMGB1 [72]. RAGE was the first identified receptor for HMGB1 in neuritis and macrophages [73]. Mounting evidence suggests that RAGE is required for HMGB1-induced injury, inflammation, and immunity and that RAGE activation is essential for this pathology [74–76]. Moreover, HMGB1 located in the extracellular space can lead to the upregulation of RAGE in multiple cell types [77, 78].

Recent studies show that the HMGB1/RAGE signaling axis might be a potential target for diseases such as diabetes [79], neurodegeneration [80], inflammation [81], and NPP [42]. Administration of an HMGB1 neutralizing antibody blocking HMGB1/RAGE signaling depressed the RAGE level and significantly alleviated mechanical allodynia in a SNL model [57]. In a rat NPP model induced by tibial nerve injury (TNI), all-thiol HMGB1 activation of nociceptive neurons was dependent on RAGE [42].* RAGE* mRNA and the protein expression in the lumbar dorsal root ganglion (DRG) were substantially increased when compared with sham injured rodents [42]. To distinguish the possible roles of RAGE in NPP, a neutralizing antibody against RAGE (RAGE Ab) was administered. RAGE Ab treatment did not abrogate pain behavior at postinjury day (PID) 7, 14, or 21, but it resulted in the reversal of mechanical hyperalgesia on PID28 [42]. Cyclophosphamide, administered intraperitoneally, caused bladder pain-like nociceptive behavior and referred hyperalgesia accompanying cystitis symptoms [64]. Tanaka et al. found that blocking HMGB1 or RAGE, using neutralizing antibodies, prevented cyclophosphamide-induced bladder pain and referred hyperalgesia [64]. Thus, these data suggest that a RAGE-triggered damage signal is involved in HMGB1 activation and may be responsible for sensory neuron sensitization and mechanical hyperalgesia associated with NPP. Furthermore, these data suggest that targeting HMGB1 or blocking RAGE might serve as a novel therapeutic strategy for the management of NPP.

### 4.2. TLR

The Toll-like receptors (TLRs) are a type I transmembrane superfamily, which is highly evolutionarily conserved in diverse species. TLRs consist of extracellular leucine-rich repeat (LRR) domains, by which pathogen-associated molecular pattern (PAMP) recognition is evoked under conditions of cytopathology [82]. TLRs are extensively expressed in innate immune cells such as macrophages and DCs, as well as in nonimmune cells such as epithelial cells and fibroblasts. Based on location, TLRs are sorted into two subfamilies including cell surface receptors (TLR-2, TLR-4, TLR-5, TLR-6, and TLR-10) and intracellular receptors (TLR-3, TLR-7, TLR-8, TLR-9, TLR-11, TLR-12, and TLR-13) [83, 84]. HMGB1 works together with TLR-2, TLR-4, and TLR-9 and then triggers the NF-*κ*B and IRF pathways, resulting in the elevated expression of inflammation factors [85].

Due to its striking role in inflammatory reactions, attention was focused on TLRs in the CNS. TLRs are expressed in both neuronal and nonneuronal cells in the CNS and contribute to both infectious and noninfectious disorders in the CNS. Following tissue insult and nerve injury, TLRs (such as TLR-2 and TLR-4) induce the activation of microglia and astrocytes and the production of proinflammatory cytokines in the spinal cord, which leads to the development and maintenance of inflammatory pain and NPP [86, 87]. Emerging evidence suggests that TLRs and their associated signaling components contribute to pain hypersensitivity and blockading of TLR signaling reduces pathological pain [88]. Since some members of the TLR subfamily are thought to function as receptors for disulfide HMGB1, HMGB1 could affect NPP by amplifying and maintaining the inflammatory response via the TLR pathway [2]. Accordingly, nerve injury-induced NPP is impaired after deletion or inhibition of TLR-2 and TLR-4 [89, 90], while application of a TLR-9 antagonist blocks tumor-induced thermal hyperalgesia [91].

In an arthritis induced murine pain model, both the levels of* HMGB1* mRNA and endochylema protein in the lumbar spinal cord were significantly increased [62]. Further investigations indicated that the pronociceptive effect of intrathecal (i.t.) injection of HMGB1 was absent in* TLR-4* deficient mice [62]. In a SNL model of NPP, Ma et al. found that HMGB1 and TLR-4 were increased at both the protein and mRNA levels [44]. Furthermore, mechanical hypersensitivity in collagen antibody-induced arthritis (CAIA) can be reversed by administration of a monoclonal neutralizing antibody against HMGB1 [62]. When using an agent that can produce anti-inflammatory effects via the inhibition of inflammatory cytokines such as IL-1*β*, IL-6, and TNF-*α*, the levels of HMGB1 and TLR-4 were downregulated and the paw mechanical withdrawal threshold (PWT) data indicated that the NPP was alleviated after treatment [44, 87]. In another study, NPP was induced in rats by chronic compacting injury of the sciatic nerve (CCI) [19]. In the CCI rat spinal cord, the mRNA and protein levels of HMGB1 and TLR-4, as well as the levels of TNF-*α* and IL-1*β*, were upregulated, and the expression of NF-*κ*B in the spinal dorsal horn was significantly increased [19]. Furthermore, the abovementioned effects were reversed by inhibition of the TLR-4, which was accompanied by a dramatic improvement in pain behaviors [19, 44]. These data suggest that the HMGB1/TLR-4 signaling pathway plays a central role in the occurrence and development of NPP, and a therapy targeting HMGB1/TLR-4 might be a novel strategy for the treatment of NPP.

However, very recently, the interaction of HMGB1 with TLRs was identified in another NPP model induced by TNI [42]. HMGB1 activation of nociceptive neurons was shown not to be dependent on TLR-2 and TLR-4 since incubation with small molecule inhibitors of TLR-2 or TLR-4 failed to alter repeated current pulse induced action potential in the presence of HMGB1 [42]. Although the results of this study are different from previous work, there are currently no additional reports on how NPP is affected by HMGB1-induced activation. More detailed studies are thus required to confirm the HMGB1/TLR interaction and to reveal its potential role in the pathogenesis of NPP.

### 4.3. Other Receptors

Besides RAGE and TLR4, NMDA receptor and CXCR4 are also involved in the HMGB1-activated inflammatory response. The disulfide forms of HMGB1 do not bind to TLR4 but can promote cytokine production through cooperation with the chemokine CXCL12 for activation of CXCR4 [32]. CXCL12 is indispensable for HMGB1-induced inflammation via CXCR4 signaling. Cooperation of HMGB1 and CXCL12 leads to stimulation of CXCR4 and recruitment of inflammatory cells around damaged cells [92]. Investigations also revealed that the NMDA receptor is involved in HMGB1-mediated neural damage [93, 94]. Disulfide HMGB1 activates and facilitates NMDA receptor-induced damage responses, such as Ca^2+^ influx and nitric oxide synthase, which leads to cell death [49]. Furthermore, using MK-801, a NMDA channel blocking agent, the effect of HMGB1 was abolished [94]. These data suggest that NMDA receptor is critical for HMGB1-mediated cell responses and might be independent of other signaling pathways in the HMGB1-activated inflammatory response.

## 5. HMGB1 as a Potential Therapeutic Target for NPP

Our recent understanding of the mechanisms and, consequently, the diagnosis of NPP has become progressively clearer in clinical practice. However, the treatment of this condition remains challenging [95]. One of the reasons for this difficulty is the diversity of mechanisms involved in NPP and its persistence [95]. Growing evidence supports a role for HMGB1 as an essential inflammatory pronociceptive factor that acts alone or cooperates with other mediators including RAGE, TLRs, TNF-*α*, and IL-1*β* and supports that HMGB1 is involved in the pathology of NPP [32, 44, 62, 72, 74]. As shown by many studies, agents (e.g., HMGB1 neutralizing antibody, HMGB1 inhibitor, and HMGB1 receptor neutralizing antibody or inhibitor) that prevent the action of HMGB1 reduce hypersensitivity in animal models of NPP [44, 72, 75, 78]. Additionally, the level of HMGB1 in samples such as serum, plasma, and cerebrospinal fluid (CSF) has been determined using Western blot or ELISA. The results indicate that HMB1 might be a promising biomarker for human disease diagnosis and therapeutic outcome [27]. However, more studies are needed to elucidate the underlying mechanisms in order to use these agents as pain-relieving drugs in human subjects.

## 6. Concluding Remarks and Future Directions

NPP still presents as a major diagnostic and therapeutic challenge despite considerable progress in the understanding of its mechanisms and the publication of a number of studies that assessed the efficacy and safety of drugs used for symptomatic treatment. HMGB1 plays an essential role in the pathogenesis of a large number of inflammatory conditions and has also been identified as a mediator of neuroinflammation. Accumulating data indicate that HMGB1 plays an important role in NPP progression. Strategies that target HMGB1 and its associated cell signaling pathways markedly depress inflammatory reactions in several models of NPP. This may lead to a promising and efficacious clinical approach for the treatment of NPP. Cell surface receptors, including RAGE, TLR, CXCR4, and NMDA receptor, are important signaling pathways in inflammatory injury. They share common ligands and signaling pathways that are involved in HMGB1-induced cellular pathology, and mounting of evidence points towards their cooperative role in the host immune response. Although much attention has been paid to the association of HMGB1 with RAGE or TLRs, there are still many unknowns underlying the association of HMGB1 with NPP. Furthermore, there are still only a few reports on* in vivo* experiments for investigating HMGB1, with even fewer on the evaluation of pathological mechanisms using either* ex vivo* or* in vitro* tests. Moreover, the mechanisms of RAGE-TLR crosstalk in response to HMGB1 are still unclear. Given the important role of these pathways in NPP, targeting them may offer new treatments for debilitating and refractory pain. Thus, further investigations are needed to better understand the pathogenesis of NPP.

## Figures and Tables

**Table 1:** The cardinal etiologies of neuropathic pain.

Classification	Frequent etiology	Reference
Disease process	Infection/inflammation, immunity, tumor infiltration, metabolic abnormalities, neurotoxicity	[2, 11, 14–16]
Therapeutic intervention	Surgery, chemotherapy, irradiation	[17, 18]
Trauma	External injury, nerve compression	[19–21]
Genetic predisposition	Inherited neurodegeneration, metabolic abnormalities, endocrine abnormalities	[22, 23]
